# Supplementary material for: Design, fabrication, characterization and reliability study of CMOS-MEMS Lorentz-force magnetometers
Source: Microsyst Nanoeng. 2022 Sep 16;8:103. doi: 10.1038/s41378-022-00423-w (PMC9481614; doi:10.1038/s41378-022-00423-w)
Supplement: Supplementary file 1 — Supplemental Material [file 41378_2022_423_MOESM1_ESM.pdf]

Supplementary Material: Design, Fabrication, Characterization and Reliability Study of CMOS-MEMS Lorentz-Force Magnetometers

J. J. Valle[a,*], J. M. Sánchez-Chiva[a,b], D. Fernández[c] and J. Madrenas[a]

[a]*Department of Electronic Engineering, Universitat Politècnica de Catalunya, Jordi Girona 1 i 3, Edifici C4, 08034 Barcelona, Spain*
[b]*Sorbonne Université, CNRS, Laboratoire de Recherche en Informatique (LIP6), UMR7606, 4 place Jussieu, 75005 Paris, France*
[c]*Institut de Física d'Altes Energies (IFAE), The Barcelona Institute of Science and Technology (BIST), Edifici Cn. Facultat Ciències Nord, Universitat Autònoma de Barcelona, 08193 Bellaterra (Barcelona), Spain*

Abstract

This file explains in detail how the Q factor of the devices was characterized as a function of pressure, resonance frequency and beam length. A simple equation that fits very well the data for two device types (lateral and vertical resonance), all the pressure points and all the device sizes/resonance frequencies, is derived based on previous works and observed phenomenology. Thermoelastic damping (TED) was found to be the limiting factor at very low pressures. Longer beams exhibited the higher Q factors.

Q factor versus pressure, resonance frequency/beam length: $Q(P, fr)$

On-wafer quality factor (Q) versus pressure (P) measurements were performed in Nitrogen (N) atmosphere at 25 °C. The results are shown in Fig. 1. They show that Q is higher the lower the pressure, reaching a saturation plateau at a pressure level that is device-dependent. This behavior obeys to the coexistence of two main damping mechanisms: air damping and intrinsic damping.

Air Damping: Approximately over a few mbar air damping is the main damping contributor. The Q-P curve follows the expected shape for an air damped resonator [1,2]. When the characteristic length of the structure (L_c) is larger than the mean free path length of the gas molecules (λ_g) the air can be modeled as a continuous viscous fluid. This is known as the fluidic regime and the Navier–Stokes equations with non-slip boundary conditions lead to [3]:

$$Q_{air} = \gamma \frac{f_r}{\mu} \quad \text{(Air damping)} \tag{1}$$

where γ is a proportionality parameter that depends on the considered geometry and f_r is the resonance frequency. The Q dependency with pressure can be introduced using a pressure-dependent artificial viscosity (μ), which has been studied for different cases (squeezed-film or shear flow, molecular or slip-flow regime, diffuse or specular gas particle reflections...)[4–8]. All these approximations have a common form, which is:

$$\mu = \frac{\mu_0}{1 + \beta K_n^m} \quad \text{(Air damping)} \tag{2}$$

where β and m are two free parameters, μ_0 is the dynamic viscosity of the gas at a specified temperature (1.81×10^{-5} Pa s at 300 K and ambient pressure), and K_n is the Knudsen number:

$$K_n = \frac{\lambda_g}{L_c} \propto \frac{T}{P} \quad \begin{cases} \lambda_g^N \sim 64\,\text{nm at } P = 1atm, T = 298K \\ \lambda_g^{Air} \sim 72\,\text{nm at } P = 1atm, T = 298K \\ L_c\text{: Characteristic length} \sim \text{air gap} \end{cases} \tag{3}$$

juanvallefraga@gmail.com (J.J. Valle); jose.sanchez_chiva@sorbonne-universite.fr (J.M. Sánchez-Chiva); dfernandez@ifae.es (D. Fernández); jordi.madrenas@upc.edu (J. Madrenas)
ORCID(s): 00000-0001-9849-7868 (J.J. Valle); 0000-0002-1101-6804 (J.M. Sánchez-Chiva); 0000-0002-1076-6697 (D. Fernández); 0000-0001-5905-9179 (J. Madrenas)

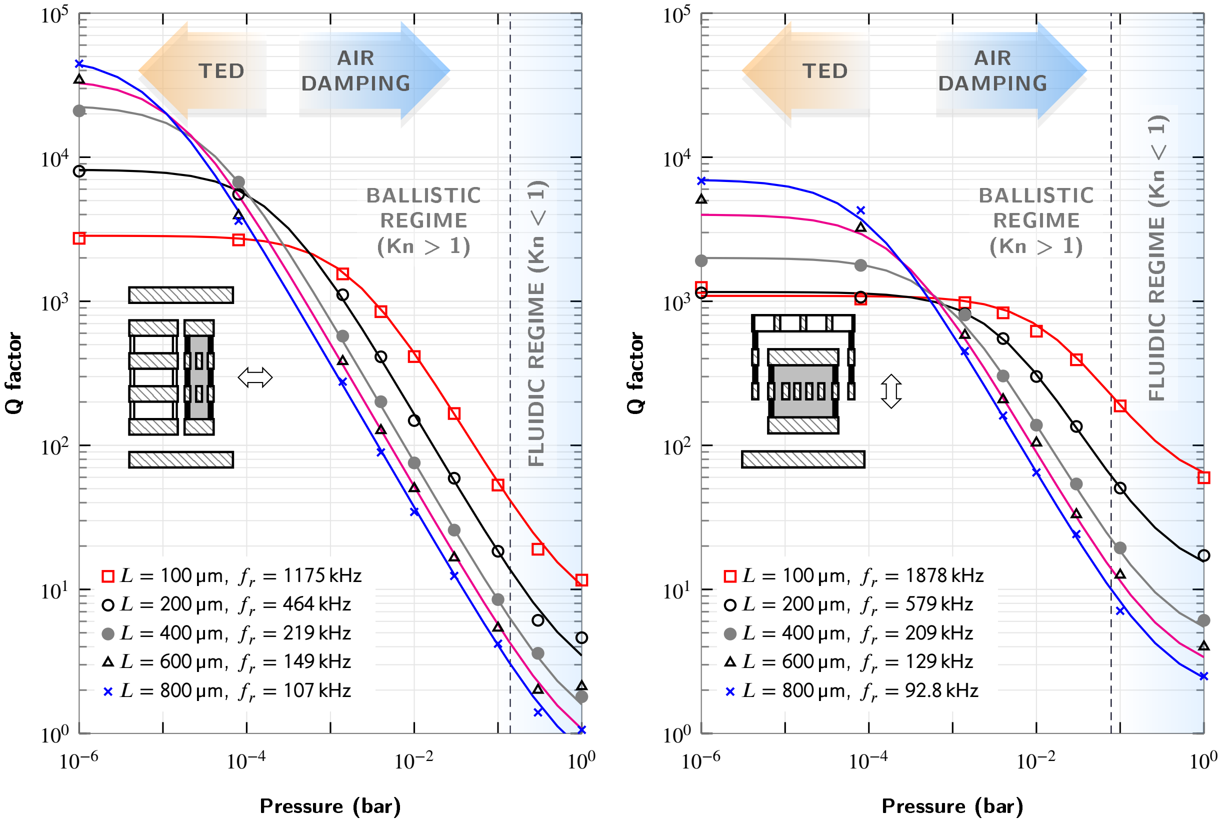

(a) Z device: Beam width = 1.5 µm, beam height = 5 µm, lateral gap = 0.35 µm.

(b) XY device: Beam width = 3 µm, beam height = 3.53 µm, vertical gap = 0.9 µm, lateral gap = 0.5 µm.

Figure 1: Measured Q versus length or f_r and pressure. The continuous lines where obtained with Eq. (8).

According to the Kinetic theory of gases λ_g is proportional to T/P. Remarkably, the artificial viscosity approach works reasonably well even when $K_n > 1$ and therefore the dissipation is caused not by viscous forces but by the impact of noninteracting gas molecules. This is called the ballistic or free molecular flow regime.

Intrinsic Damping: Intrinsic damping generally represents the Q factor upper limit at sufficiently low pressure. It arises from relaxation loss mechanisms within the resonating structure itself[2]. The better known example may be thermoelastic damping (TED), which is an absolute lower bound on intrinsic damping, but friction loss mechanisms like surface loss or phase boundary slipping in multilayer structures should also be considered in CMOS-MEMS structures. Friction loss mechanisms are a ubiquitous phenomenon and, along with TED, are best described by the Zener's anelastic relaxation theory[9,10]. In this theory, the Q factor resultant from n intrinsic damping mechanisms would be given by:

$$Q_{intrinsic}^{-1} = \sum_{i=1}^{n} \Delta_i \frac{f_r/f_{Di}}{1+(f_r/f_{Di})^2} \tag{4}$$

where Δ_i is the relaxation strength and $f_{Di} = 1/(2\pi\tau_i)$ is the Debye frequency associated with the relaxation time of the ith mechanism (τ_i). Generally, one mechanism is dominant and it is sufficient to consider $n = 1$. The minimum Q factor occurs when the vibration is at the Debye frequency of the dominant one. Depending on whether f_r is well

	L_c(nm)	γ	f_c(MHz)
Z devices	350	4.4×10^{-11}	≈ 1.6
Z devices*	350	6.8×10^{-11}	≈ 2.3
Z devices	500	8.3×10^{-11}	≈ 2.0
Z devices	1000	26×10^{-11}	≈ 2.0
XY devices**	900	35×10^{-11}	≈ 3.0

*Sensing electrode is hollow between layers and air can flow through. **Double vertical gap of 900 nm, one of them with holes and air can flow through.

Table 1
Parameters used in Eq. (7).

below f_D (isothermal regime) or well above it (adiabatic regime) the Q dependency with f_r is the opposite:

$$Q_{intrinsic} = \begin{cases} (f_r/f_D)/\Delta & \text{if } f_r \gg f_D \text{ (Adiabatic)} \\ 2/\Delta & \text{if } f_r = f_D \text{ (Debye Peak)} \\ (f_D/f_r)/\Delta & \text{if } f_r \ll f_D \text{ (Isothermal)} \end{cases} \tag{5}$$

In the case of TED and for uniform beams[2]:

$$\Delta_{TED} = \frac{E\alpha^2 T}{\rho C_p} \quad \text{and} \quad f_D^{TED} = \frac{\pi^2 k}{t^2 \rho C p} \tag{6}$$

where E is the Young's Modulus, α the thermal expansion coefficient, ρ the density, C_p the specific heat, k the thermal conduction coefficient and t the beam thickness.

Q factor characterization: Our data clearly shows a $Q \propto P^{-1}$ dependency in the ballistic regime, which implies $m = 1$ in Eq. (2), close to most formulas in Veijola et al.[6], Li and Hughes[8]. Most authors use Veijola's formula with $m = 1.159$, intended for diffusely rejecting identical surfaces[6], but it does not work well in our case ($3 - 5$ µm wide and $100 - 800$ µm long BEOL CMOS beams with $0.35 - 1.00$ µm gaps where both slide and squeeze film damping take place).

The β value models $Q(P)$ in the fluidic regime ($K_n \leq 1$). Typically, it may range from $\beta = 2$ for shear flow[5] to values not usually higher than 10, as shown in Li and Hughes[8]. In our case, $\beta = 5$ worked reasonably well. The proportionality factor between Q and f_r/μ in Eq. (1) defines the slope of the curve in the ballistic regime. It turns out to be $\gamma = 6.80 \times 10^{-11}$ Pa s^2 for the z devices and $\gamma = 3.50 \times 10^{-11}$ Pa s^2 for the vertical devices.

The shortest devices showed Q factors up to 30 % higher than initially expected in the air-damped region, according to their resonance frequency and Eq. (1). We think it may be caused by the air not being able to escape from the closing gap fast enough and starting to behave more like a spring and less like a damper. In this case, the damping coefficient will change with frequency f_r as $\propto 1/(1 + f_r^2/f_c^2)$, where f_c is the cut-off frequency[11]. The approximate cut-off frequency that best fitted the data was $f_c \approx 2.3$ MHz for the z device and $f_c \approx 3.0$ MHz for the vertical device.

After adding all the discussed corrections to Eq. (1), the Q factor due to air damping is, finally:

$$Q_{air} = \gamma \frac{f_r}{\mu_0} \left(1 + \frac{5\lambda_g}{L_c}\right)\left(1 + \frac{f_r^2}{f_c^2}\right) \tag{7}$$

where the pressure dependence is contained in $\lambda_g \propto T/P$ and $\mu_0 = f(T)$ may be considered independent of P. Table 1 summarizes the parameters used for the 2 cases represented in Fig. 1 (highlighted), and also provides experimental data for three additional cases.

At low pressures, another damping mechanism becomes the dominant one and the measured quality factors reach a plateau (see Fig. 1). We have plotted the measured Q factor as a function of the resonant frequency at 1 µbar in Fig. 2 in order to analyze the dominant damping mechanism. The z devices (see plateaus in Fig. 1a and circular data points in Fig. 2) operate in the isothermal region, where $Q \propto 1/f_r$, just the inverse proportionality of that found in the

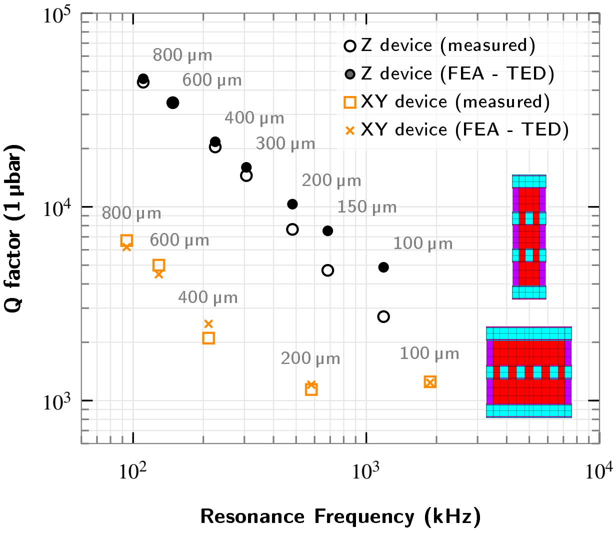

Figure 2: Measured Q versus frequency at $P = 1$ µbar and predicted Q factor with finite element analysis (FEA) caused by thermoelastic damping (TED). Meshed cross-sections of the two types of devices also shown.

L =	100 µm	200 µm	400 µm	800 µm
Z devices Q_{TED} =	2850	8200	23000	50000
XY devices Q_{TED} =	1090	1160	2000	7000

Table 2
Quality factors due to TED (Q_{TED}) used in Eq. (8).

air-damped region. On the other hand, the shorter xy devices (Fig. 1b and square data points around 1MHz in Fig. 2) seem to operate close to their Debye frequency given than the Q factor does not depend that much on the vibration frequency.

Duwel et al.[12] has shown that TED is an important loss mechanism for flexural modes. However, Prabhakar and Vengallatorer observed in Prabhakar and Vengallatore[13] that internal friction is much higher than TED when $f_r < 1$ MHz in some bilayer structures. Given that CMOS-MEMS devices are multilayered and more complex than theirs, intrinsic friction losses might be important. However, finite element analysis (FEA) carried out by us predicted Q factors due to TED very similar to the measured ones for both types of devices. The simulated TED Debye frequency for the vertical device (0.8 MHz) is substantially smaller than for the lateral one (larger than 2 MHz). This explains the higher Q factors for the longest devices at low pressure. However, all the CMOS BEOL layers must be included in the FEA model (see cross-sections in Fig. 2), even the adhesion and antireflective coatings (Titanium and Titanium Nitride) in order to perform sufficiently accurate predictions. These layers play an important role because they have a low thermal conductance which decreases the associated TED Debye frequency and this determines greatly the simulated Q factor. Also, stress in the beams was included in the simulations given its importance in highly stressed structures[2,14]. The only deviation from simulations takes place in the shortest lateral devices, for which the TED Q factor is overestimated. It might be evidence of another damping mechanism that we have not identified.

The total Q factor is, therefore:

$$\frac{1}{Q_{total}} = \frac{1}{Q_{air}} + \frac{1}{Q_{TED}} \tag{8}$$

All the solid lines in Fig. 1 were generated with Eq. (8). The value used for Q_{TED} is shown in Table 2 and was deduced from the measured data, rather than the simulation because, as already mentioned, there is some disagreement for the shortest lateral devices at 1 µbar. With that exception, Eq. (8) fits very well the measured data.

Author information

Corresponding author

Correspondence to Juan Valle.

References

[1] Maria F Pantano, Leonardo Pagnotta, and Salvatore Nigro. A numerical study of squeeze-film damping in MEMS-based structures including rarefaction effects. *Frattura ed integrita strutturale*, 7(23):103–113, 2013. doi: 10.3221/IGF-ESIS.23.11. URL https://www.fracturae.com/index.php/fis/article/view/168.

[2] Silvan Schmid, Luis Guillermo Villanueva, and Michael Lee Roukes. *Fundamentals of nanomechanical resonators*, volume 49. Springer, 2016. ISBN 978-3-319-28691-4. doi: 10.1007/978-3-319-28691-4. URL https://www.springer.com/gp/book/9783319286891?utm_campaign=bookpage_about_buyonpublisherssite&utm_medium=referral&utm_source=springerlink#otherversion=9783319286914.

[3] Adi Minikes, Izhak Bucher, and Gal Avivi. Damping of a micro-resonator torsion mirror in rarefied gas ambient. *Journal of Micromechanics and Microengineering*, 15(9):1762, 2005. doi: 10.1088/0960-1317/15/9/019. URL https://iopscience.iop.org/article/10.1088/0960-1317/15/9/019.

[4] Ali Beskok and George Em Karniadakis. Report: a model for flows in channels, pipes, and ducts at micro and nano scales. *Microscale thermophysical engineering*, 3(1):43–77, 1999. doi: 10.1080/108939599199864. URL https://www.tandfonline.com/doi/abs/10.1080/108939599199864.

[5] Albert Burgdorfer. The influence of the molecular mean free path on the performance of hydrodynamic gas lubricated bearings. *Journal of Basic Engineering*, 81(1):94–98, 1959. doi: 10.1115/1.4008375. URL https://asmedigitalcollection.asme.org/fluidsengineering/article-abstract/81/1/94/368450/The-Influence-of-the-Molecular-Mean-Free-Path-on?redirectedFrom=fulltext.

[6] Timo Veijola, Heikki Kuisma, and Juha Lahdenperä. The influence of gas-surface interaction on gas-film damping in a silicon accelerometer. *Sensors and Actuators A: Physical*, 66(1):83 – 92, 1998. ISSN 0924-4247. doi: 10.1016/S0924-4247(97)01732-9. URL http://www.sciencedirect.com/science/article/pii/S0924424797017329.

[7] Timo Veijola, Heikki Kuisma, Juha Lahdenperä, and Tapani Ryhänen. Equivalent-circuit model of the squeezed gas film in a silicon accelerometer. *Sensors and Actuators A: Physical*, 48(3):239 – 248, 1995. ISSN 0924-4247. doi: 10.1016/0924-4247(95)00995-7. URL http://www.sciencedirect.com/science/article/pii/0924424795009957.

[8] Gary X Li and Henry G Hughes. Review of viscous damping in micromachined structures. In Eric Peeters and Oliver Paul, editors, *Micromachined Devices and Components VI*, volume 4176, pages 30–46. International Society for Optics and Photonics, SPIE, 2000. doi: 10.1117/12.395618. URL https://doi.org/10.1117/12.395618.

[9] Clarence Zener. Internal friction in solids I. Theory of internal friction in reeds. *Phys. Rev.*, 52:230–235, Aug 1937. doi: 10.1103/PhysRev.52.230. URL https://link.aps.org/doi/10.1103/PhysRev.52.230.

[10] Clarence Zener. Internal friction in solids II. General theory of thermoelastic internal friction. *Phys. Rev.*, 53:90–99, Jan 1938. doi: 10.1103/PhysRev.53.90. URL https://link.aps.org/doi/10.1103/PhysRev.53.90.

[11] M.A.G. Suijlen, J.J. Koning, M.A.J. van Gils, and H.C.W. Beijerinck. Squeeze film damping in the free molecular flow regime with full thermal accommodation. *Sensors and Actuators A: Physical*, 156(1):171 – 179, 2009. ISSN 0924-4247. doi: 10.1016/j.sna.2009.03.025. URL http://www.sciencedirect.com/science/article/pii/S0924424709001691. Eurosensors XXII, 2008.

[12] Amy Duwel, Rob N Candler, Thomas W Kenny, and Mathew Varghese. Engineering MEMS resonators with low thermoelastic damping. *Journal of microelectromechanical systems*, 15(6):1437–1445, 2006. doi: 10.1109/JMEMS.2006.883573. URL https://ieeexplore.ieee.org/document/4020265.

[13] Sairam Prabhakar and Srikar Vengallatore. Thermoelastic damping in bilayered micromechanical beam resonators. *Journal of Micromechanics and Microengineering*, 17(3):532, 2007. doi: 10.1088/0960-1317/17/3/016. URL https://iopscience.iop.org/article/10.1088/0960-1317/17/3/016.

[14] G. Cagnoli, J. Hough, D. DeBra, M.M. Fejer, E. Gustafson, S. Rowan, and V. Mitrofanov. Damping dilution factor for a pendulum in an interferometric gravitational waves detector. *Physics Letters A*, 272(1):39 – 45, 2000. ISSN 0375-9601. doi: 10.1016/S0375-9601(00)00411-4. URL http://www.sciencedirect.com/science/article/pii/S0375960100004114.
